# DIC-like syndrome in a post-pre-eclampsia birth in a premature infant in a peri-COVID scenario

**DOI:** 10.1515/crpm-2023-0016

**Published:** 2023-10-25

**Authors:** Taha F. Hassan, Ryan D. Morgan, Akshay Raghuram, Benedicto C. Baronia

**Affiliations:** School of Medicine, Texas Tech Health Sciences Center, Lubbock, TX, USA; Surgery, Texas Tech Health Sciences Center University Medical Hospital, Lubbock, TX, USA

**Keywords:** ICU, neonate care, pre-eclampsia, VPS shunt

## Abstract

**Objectives:**

This article outlines an unusual presentation of a premature infant born from a pre-eclamptic mother born with a presentation resembling a disseminated intravascular coagulation syndrome.

**Case presentation:**

Pregnancy-induced hypertension, also known as pre-eclampsia, and premature birth pose significant risks to neonates, making the fetus more susceptible to immunodeficiencies and coagulopathies. This article highlights a premature infant born to a pre-eclamptic mother with multiple complications. Our case involved jaundice, neonatal meningitis, thrombocytopenia, leukopenia, neutropenia, hemorrhage, apnea, gastrointestinal defects, and periventricular leukomalacia. Often these complications are seen immediately after birth; these symptoms may present after a certain amount of time lapses if the neonates if afflicted with malignancy or a viral, fungal, or bacterial infection. Here we describe the case of a premature neonate born to a preeclamptic mother that experienced these complications one day after her birth.

**Conclusions:**

This is the first known case of an infant experiencing a “DIC-like” syndrome without any diagnosis of a primary hematological malignancy or infection after a certain amount of time had lapsed since her birth. As complications in premature infants as well as those from pre-eclamptic mothers are common, this case report highlights a successful model of care. We also explore the effect of a peri-COVID setting on the presentation of this patient, as similar cases have occurred post-COVID-19.

## Introduction

Pre-eclampsia is defined as hypertension that can occur during pregnancy [1]. While some patients may possess genetic predispositions towards developing pre-eclampsia, the underlying pathophysiology is thought to originate from aberrant interactions with the placenta [1]. Pre-eclampsia can also affect the neonate, inducing a pro-inflammatory state and disrupting fetal hematopoiesis, inducing coagulopathies and weakened immunity [2]. A study by Davies et al. found that pre-eclampsia is often an indicator and contributor to premature delivery, most likely due to the risk of a limited blood supply limiting fetal growth [3, 4]. Premature infants are more susceptible to intellectual and physical development complications and have greater hospital admission rates than full-term delivered neonates [3].

Several complications have been identified in infants delivered prematurely and those born from pre-eclamptic mothers. These infants present with a “disseminated intravascular coagulation (DIC)-like” syndrome, characterized by various coagulopathies, sepsis, and end-organ involvement without qualifying for a diagnosis of DIC. However, in similar cases, most of these complications present immediately after birth [5]. Here, we report the case of a premature infant from a pre-eclamptic mother that had a delayed onset of “DIC-like” symptoms.

## Case presentation

This case outlined a female newborn with a gestational age of 29 weeks who required premature induction due to severe pre-eclampsia. The mother was an otherwise healthy 24-year-old who received appropriate prenatal care but experienced severe abdominal pain and dizziness prior to giving birth. The patient was born in stable condition at 1.01 kg, requiring a heating mattress for hypothermia. Due to concerns about developing prematurity, respiratory insufficiency, premature retinopathy, jaundice, and hyperbilirubinemia, the patient was transferred within an hour of birth to the neonatal intensive care unit (NICU) for further management, which included infectious screening.

**Figure 1:**
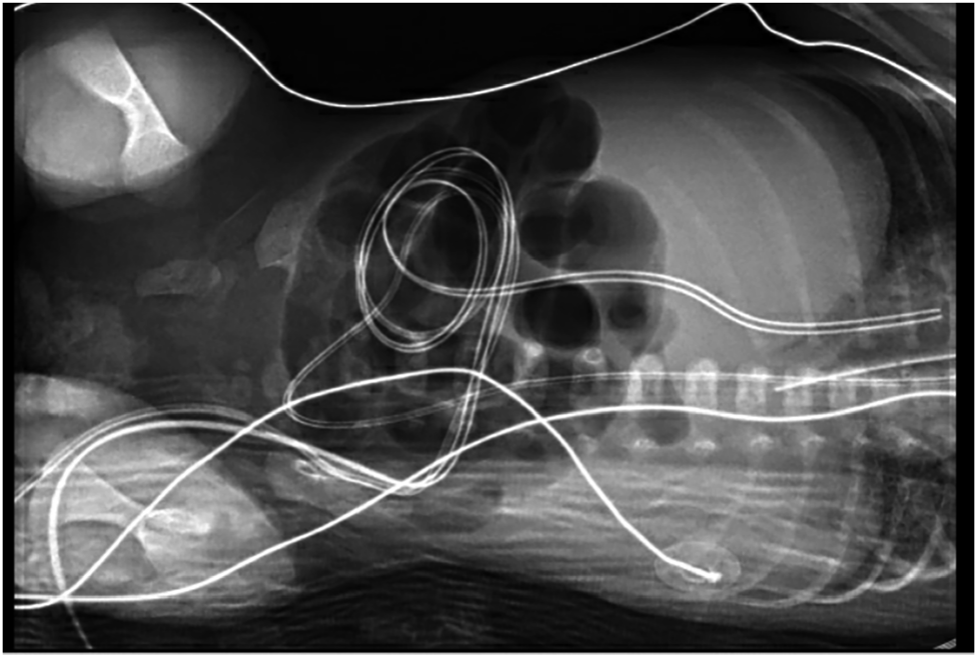
Radiographic imaging revealed that there is no evidence of free air in the peritoneal cavity. There was no evidence of portal physical exam findings consisted of generalized edema that seemed to worsen over the next five days. On DL 8, she developed hemolytic uremia syndrome (HUS) with a right grade three hemorrhage and left grade two hemorrhage in the cerebral ventricles and mild prominence of the right lateral ventricle (Figure 2). Her worsening edema manifested itself as a round and full abdomen and a rounded head. Abdominal ultrasound on DL 9 found trace ascites and pelviectasis venous gas.

On DL (day of life) two, the baby exhibited pale, yellow mottled skin and persistent apneic/bradycardic episodes. The patient was administered an IV infusion of epinephrine and dopamine. Radiographic imaging was performed which revealed minimal levels of surfactant deficiency, a typical abdominal bowel gas pattern (Figure 1), and no presence of a pneumothorax. Arterial blood gas (ABG) tests were positive for metabolic acidosis and hyperglycemia. The neonate was administered one dose of protectant alfa, NS bolus 10 mL/kg, and a decreased glucose infusion rate. White blood cell count (WBC) (1.58 × 10^9^/L), platelets (99 × 10^9^/L), and absolute neutrophil count (ANC) (0.147 K/μL) were decreased on a complete blood count (CBC) taken at this time. Additionally, she was placed on a regimen of ampicillin and gentamicin for meningitis prophylaxis due to neutropenia.

**Figure 2:**
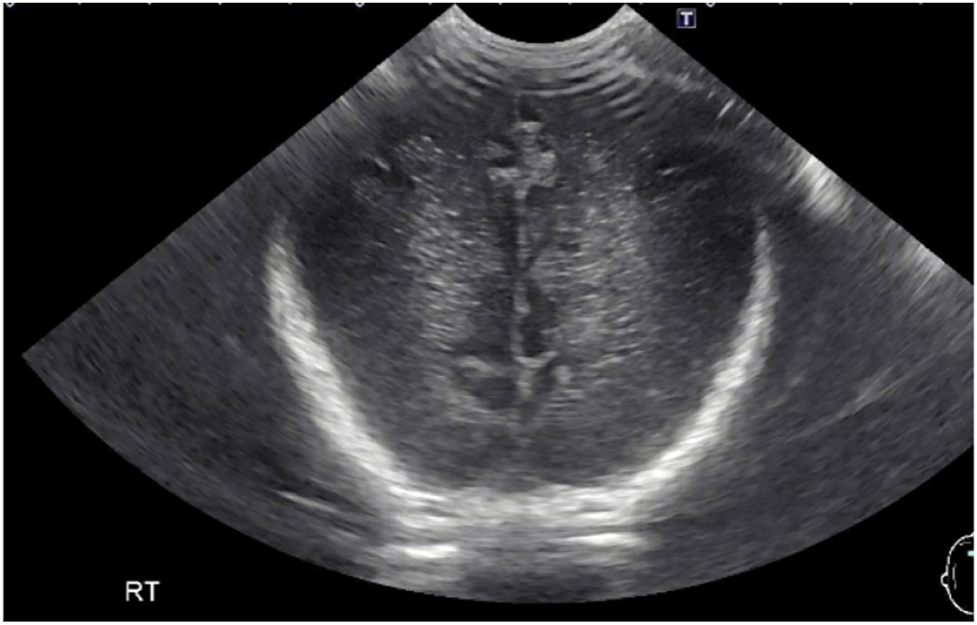
Cranial ultrasound revealed hemorrhage in the caudothalamic groove along with intraventricular extension bilaterally. There was mild prominence of the right lateral ventricle. Overall findings are compatible with right grade 3 hemorrhage and left grade 2 hemorrhage.

On DL 3, the patient developed direct hyperbilirubinemia with elevated total serum bilirubin, 5.5 mg/dL, and phototherapy was initiated. She received platelet transfusions and packed red blood cell transfusions as needed for her thrombocytopenia and microcytic anemia. in bilateral kidneys. The patient’s WBC (21.05 × 10^9^/L) and ANC (16.802 K/μL) improved from previous lab findings, although both levels were still lower than the threshold. Direct bilirubin levels were still significantly greater than the normal range. On DL 10, the patient experienced a decreased range of motion in the upper extremities; however, the patient’s HUS and color had improved. On DL 15, the patient was also noted for seizure activity in the morning with twitching arms, legs, and face. After a single dose of phenobarbital, her seizure activity ceased.

The patient’s lab results on DL 16 found low T4 (3.49 mcg/dL) and normal thyroid stimulating hormone (TSH) (4.07 mcIntUnit/mL). She was subsequently diagnosed with severe combined immunodeficiency disease (SCID), also indicated by soft T cell receptor excision circles (TRECs). Neuroimaging revealed stable enlargement of lateral ventricles with an interval evolution of an intraventricular clot without periventricular leukomalacia.

**Figure 3:**
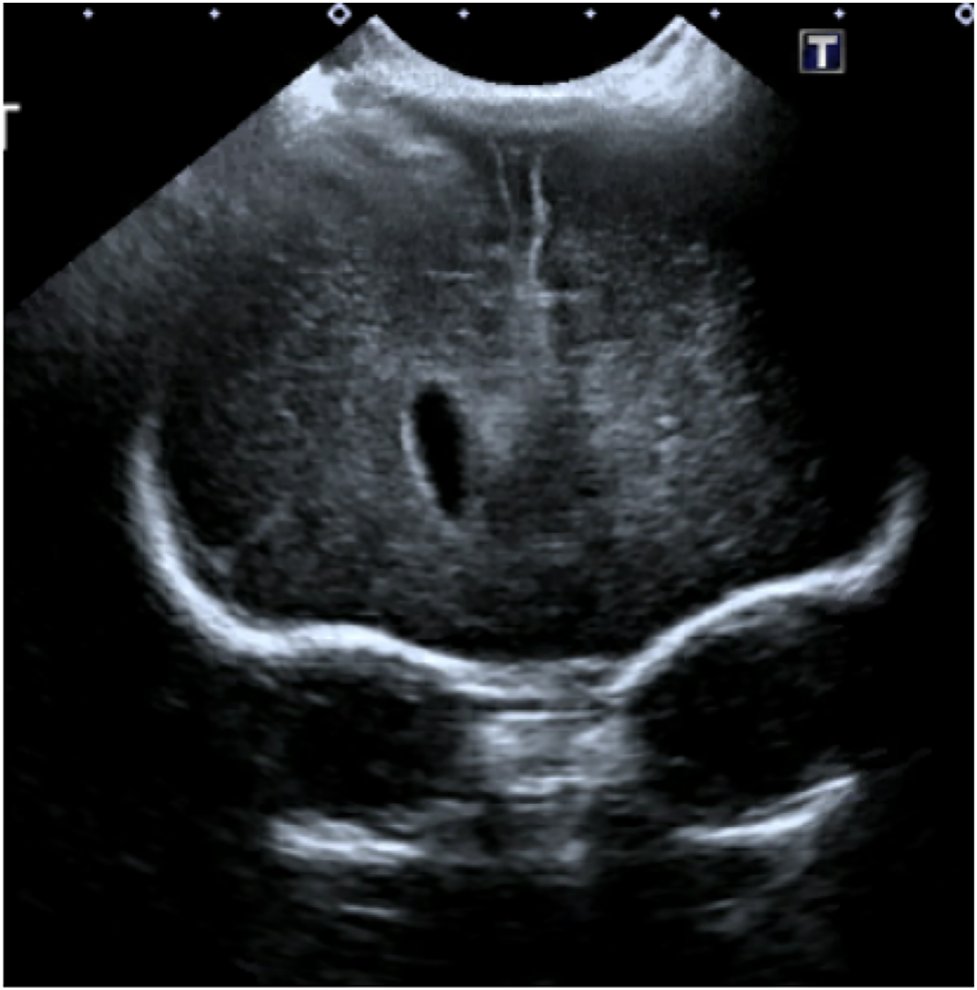
Head CT showed no abnormalities in extra-axial fluid space, periventricular white matter, and midline and posterior fossa structures. The ventricles were mildly enlarged but stable from prior examinations. Imaging also showed stable bilateral germinal matrix hemorrhages.

A cranial ultrasound on PAD 20 indicated severe intracranial stigmata of prematurity, massive intraventricular hemorrhage, obstructive hydrocephalus, and hemorrhagic necrosis of the proper temporal periventricular white matter. Hepatitis A IgG results were also reactive with grade 1 bilateral hydronephrosis on abdominal ultrasound. Pediatric neurosurgery tapped cerebrospinal fluid (CSF) on DL 27 and removed 13 mL, which came back positive for *Escherichia coli*, and the patient was initiated on Cefepime. Additionally, a right frontal extra ventricular drainage (EVD) was placed to treat the patient’s obstructive hydrocephalus. On DL 28, she developed increased twitching and was loaded on fosphenytoin; however, EEG activity could not be obtained due to scalp lesions. Direct bilirubin went down to 5.6 mg/dL on, and the patient remained on ursodiol to prevent cholesterol gallstones. The patient continued to twitch, so levetiracetam was added to the fosphenytoin. By DL 35, CSF cultures were negative for *E. coli* and CT indicated resolution of acute intracranial hemorrhage and stable ventricular size and configuration (Figure 3).

**Figure 4:**
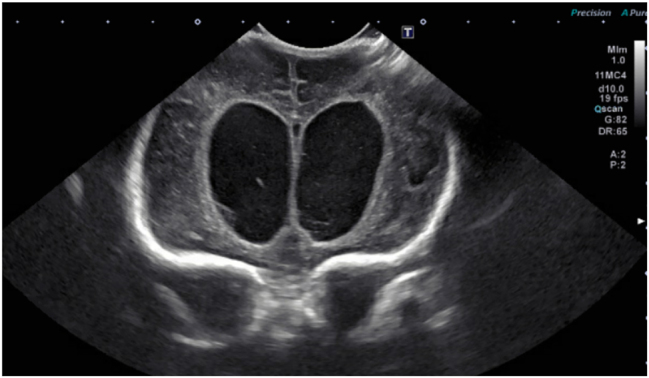
Cranial ultrasound displayed lateral ventricles are enlarged with biparietal diameter measuring approximately 3.9 cm. On the prior exam, this measured 3.6 cm. Intraventricular clot is seen in the bilateral ventricles. There was no significant midline shift. There was an interval evolution of intraventricular clot without extra-axial fluid collection.

On DL 42, cranial ultrasound noted internal enlargement of the lateral ventricle, a reoccurring intraventricular clot with periventricular leukomalacia changes, and encephalomalacia seen in the right frontal lobe (Figure 4). EVD was discontinued on DL 47 and levetiracetam was discontinued on DL 55. A rapid MRI 58 days after birth noted some diffusion changes, but her overall condition was improved compared to previous imaging. However, multi-cystic hydrocephalus was now found on the MRI. Her most recent direct bilirubin lab result is almost within upper limits of normal, and ursodiol was discontinued on DL 59. Despite requiring occasional platelet and red blood cell transfusions, as of DL 78, she is in stable condition released on a high-risk discharge. The patient will continue to follow-up with primary care as needed.

## Discussion

Pre-eclampsia and premature delivery have been shown to exert adverse effects on neonates. Pre-eclampsia, diagnosed by a systolic blood pressure >140 mmHg or a diastolic blood pressure >90 mmHg, alters the fetus’s intrauterine environment, resulting in an increased rate of adverse outcomes [6]. Negative consequences associated with pre-eclampsia include, but are not limited to, neonatal thrombocytopenia (platelet count <100,000/µL), intracranial/periventricular hemorrhage, and hypoxic-ischemic encephalopathy/periventricular leukomalacia [7]. Preterm neonates, or babies born before 37 weeks of pregnancy, may also present with a greater likelihood of adverse outcomes, such as greater mortality rates, neonatal respiratory distress syndrome, bronchopulmonary dysplasia, chronic lung disease, apnea, gastrointestinal defects, immunodeficiency, systemic hematological deficiencies, neural defects, hearing impairments, vision defects, intracranial and intraventricular hemorrhage, white matter injury, and periventricular leukomalacia [8]. However, the pathophysiological mechanisms regarding the manifestation of defects in pre-eclampsia and premature infants are not entirely understood, and it is currently of great interest for ongoing research.

Certain neonatal complications, such as hematological disorders, typically occur immediately post-partum. These complications can progress and cause other downstream issues [6]. Although our patient tested negative for an elevated D-dimer test, her symptoms resemble that of disseminated intravascular coagulation (DIC). DIC is a life-threatening secondary disease that alters the coagulation cascade by forming micro clots in the peripheral vasculature. This precipitates bleeding due to the consumption of the clotting [9]. DIC is often secondary, stemming from obstetric complications such as pre-eclampsia or premature delivery. Neonates are at a greater risk for DIC because they all have developmental coagulopathy or slightly altered hemostasis, which puts them at risk whenever their body is under stress by other conditions [9]. DIC can cause multi-organ damage due to hemorrhage and ischemia, causing complications such as respiratory distress, jaundice, liver failure, ecchymosis, and renal failure [10]. When DIC arises due to obstetric complications, it typically presents immediately after birth, whereas our patient began presenting with DIC-like symptoms one day after birth. Cases where these symptoms do not present immediately after birth are usually due to autoimmune diseases, myeloproliferative disorders, or infection in neonates. However, in our patient, bacterial infection occurred after the onset of complications such as hyperbilirubinemia, respiratory distress, neutropenia, and thrombocytopenia [11].

It is also important to note that this patient was born in the summer of 2022 following the COVID-19 pandemic and release of the immunizations. Fell et al. found no connection between the number of preterm births, stillbirths, or babies that are small for gestational age and maternal COVID-19 vaccination status [12]. However, several murine studies have shown that non-neutralizing antibodies from the COVID-19 vaccine that are transferred during gestation enhance the severity of neonatal complications, such as lymphopenia and thrombocytopenia [13], [14], [15]. Non-neutralizing antibodies enhance viral entry into host cells [13]. The mother, in this case, had previously received the COVID-19 vaccine. Although we cannot confirm any association or causation between the COVID-19 vaccination and neonatal complications, our patient’s presentation may be connected to the vaccination status of her mother before pregnancy.

Although many of the symptoms presented in this patient are commonly attributed to prematurity and pre-eclampsia, they are typically encountered immediately after birth. In contrast, our patient’s symptoms began manifesting one day after delivery. To our knowledge, all previous idiopathic presentations of a DIC-like syndrome developed due to an infection, autoimmune disorder, or myeloproliferative disease. This is the first known case with an idiopathic presentation of a DIC-like syndrome one day following birth with the lack of a primary diagnosis.
